# Exploring the Impact of Covid-19-Related Perceptions on Psychological Distress and Quality of Life in an International Gastrointestinal Cohort Over Time Guided by the Common Sense Model

**DOI:** 10.1007/s10880-023-09937-5

**Published:** 2023-01-24

**Authors:** Simon R. Knowles, Stephan P. Möller, Andreas Stengel, Antonina Mikocka-Walus, Nuno Ferreira, Inês A. Trindade, Anna Mokrowiecka, Johan Burisch, Manuel Barreiro-de Acosta, Charles N. Bernstein, Bobby Lo, David Skvarc

**Affiliations:** 1 Department of Psychological Sciences, Faculty of Health, Arts and Design, Swinburne University of Technology, Melbourne, Australia; 2 Department of Psychosomatic Medicine and Psychotherapy, University Hospital Tübingen, Tübingen, Germany; 3 Charité Center for Internal Medicine and Dermatology, Department for Psychosomatic Medicine, Charité-Universit¨Atsmedizin Berlin, Corporate Member of Freie Universität Berlin, Humboldt-Universität Zu Berlin, and Berlin Institute of Health, Berlin, Germany; 4 School of Psychology, Deakin University Geelong, Geelong, Victoria Australia; 5 School of Humanities and Social Sciences, University of Nicosia, Nicosia, Cyprus; 6 Department of Molecular and Clinical Medicine, Institute of Medicine, Sahlgrenska Academy, University of Gothenburg, Gothenburg, Sweden; 7 Center for Research in Neuropsychology and Cognitive and Behavioural Intervention (CINEICC), Faculty of Psychology and Education Sciences, University of Coimbra, Coimbra, Portugal; 8 Department of Digestive Tract Diseases, Medical University of Lodz, Lodz, Poland; 9 Gastrounit, Medical Division, Copenhagen University Hospital - Amager and Hvidovre, University of Copenhagen, Hvidovre, Denmark; 10 Copenhagen Center for Inflammatory Bowel Disease in Children, Adolescents and Adults, Copenhagen University Hospital - Amager and Hvidovre, Hvidovre Hospital, Hvidovre, Denmark; 11 IBD Unit. Gastroenterology, University Hospital of Santiago de Compostela, A Coruña, Spain; 12 University of Manitoba IBD Clinical and Research Centre and Department of Medicine, Max Rady College of Medicine, Rady Faculty of Health Sciences, University of Manitoba, Winnipeg, Canada

**Keywords:** Common sense model, Cross-lagged panel model, Gastrointestinal, COVID-19, quality of life

## Abstract

The aim of this longitudinal study was to examine changes in COVID-19 and illness-related perceptions, gastrointestinal symptoms, coping, catastrophising, psychological distress, and QoL during the COVID-19 pandemic. A total of 831 adults with a gastrointestinal condition completed an online questionnaire at baseline (May—October 2020). Of those, 270 (32.5%) participants (85.2% female, mean age = 47.3 years) provided follow-up data (March—May 2021). Repeated-measures multiple analysis of variance and a cross-lagged panel model were used to test the study hypotheses. Gastrointestinal symptoms and COVID-19 perceptions at follow-up were strongly predicted by their baseline values, while illness perceptions were predicted by baseline gastrointestinal symptoms. Cross-lagged relationships indicated a reciprocal relationship between gastrointestinal symptoms and psychological distress. Moreover, gastrointestinal symptoms had substantial predictive utility, strongly predicting future gastrointestinal symptoms, and to a lesser extent, more negative illness perceptions, greater psychological distress, and greater use of adaptive coping strategies across time.

## Introduction

Along with their high prevalence, gastrointestinal (GI) conditions are known to cause significant burden and psychological distress. Based on a recent global prevalence study, Sperber and colleagues (Sperber et al., 2021) found that at least 1 in 4 individuals live with a GI condition. Along with their high prevalence, research also indicates that at least 20% and 25% of GI cohorts report comorbid depression and anxiety, respectively (Mikocka- Walus et al., 2019). Moreover, these rates of psychological distress are frequently higher compared to healthy controls (Clappison et al., 2020; Mikocka-Walus et al., 2016; Zamani et al., 2019). The burden of GI symptoms and psychological comorbidity has also been linked to poorer quality of life in multiple GI conditions (e.g., irritable bowel syndrome, inflammatory bowel disease, coeliac disease, and gastroparesis), including when compared to healthy controls (Cassar et al., 2020; Knowles et al., 2018a, 2018b; Knowles et al., 2018a, 2018b; Moller et al., 2021; Quigley & Hungin, 2005; Woodhouse et al., 2017). This burden has been amplified by the current COVID-19 pandemic.

SARS-Cov-2, the virus responsible for the coronavirus disease 2019 (COVID-19), has to date been confirmed in over 275 million people, leading to millions of deaths around the globe (ECDC, 2021). The COVID-19 pandemic and the resulting severe lockdown measures have caused significant disruptions to everyday life. Poorer mental health outcomes in the general population, compared to historical norms for generalised anxiety and depression, have been reported internationally (Nelson et al., 2020). These have been linked to prolonged isolation (Lee et al., 2020; Smith & Lim, 2020; Wong et al., 2020), fear of contracting SARS-Cov-2, as well as uncertainty around employment, and increased domestic violence (IASC, 2020).

Having a chronic disease has been associated with an increased risk of severe COVID-19 infection and death (Sanyaolu et al., 2020; Wang et al., 2021; Zheng et al., 2020). Patients with common GI disorders have been found to report more GI symptoms during the COVID-19 pandemic and increased medication and healthcare utilization than at other times (Gubatan et al., 2021). While specific vulnerabilities to COVID-19 vary between GI disorders, given their different aetiologies and treatments, there is an increased risk of mental illness for those with chronic GI disorders compared to healthy controls in general (Clappison et al., 2020; Mikocka-Walus et al., 2016; Zamani et al., 2019), resulting in a vulnerability to mental health deterioration at the time of severe and ongoing stress such as major disasters (Murphy et al., 2020; Rahman et al., 2020). It is, therefore, unsurprising that those with a pre-existing mental comorbidity had a higher risk of GI symptom deterioration during the pandemic (Oshima et al., 2021), while fear of COVID-19, illness perceptions, coping style and distress were found to mediate the relationship between GI symptoms and quality of life in a sample with mixed GI diagnoses (B. ). Similarly, GI symptoms moderated the association between social isolation and psychological distress at the time of the COVID-19 pandemic (Mikocka-Walus, Skvarc, de Acosta, et al., 2021). At the same time, the relationship between product shortages and psychological distress was moderated by COVID-19 fear (Mikocka-Walus, Skvarc, van Tilburg, et al., 2021).

The process of illness adaptation is complex, however understanding the processes that influence adaptation is essential so that psychological interventions can be more effective. One of the most researched and well-established theoretical models of illness adjustment is the common sense model (CSM; Leventhal et al., 1992). The CSM purports that an individual’s adjustment to illness is influenced by a dynamic interplay of factors. Specifically, the CSM suggests that the impact of illness related symptoms (e.g., GI pain) on outcomes (e.g., psychological distress, quality of life [QoL]) is influenced by illness perceptions (e.g., how controllable it is, how long it will continue, how much impact it has on one’s emotions), and coping styles (e.g., emotional avoidance, seeking support). To date, the CSM has been successfully applied across many chronic illness cohorts to explore illness adaptation and its impact on patient reported outcomes (e.g., QoL; for review, see Hagger et al., 2017; Hagger & Orbell, 2021), including GI conditions—inflammatory bowel disease (Bree ; Bree ), irritable bowel syndrome (Knowles et al., 2017), coeliac disease (Möller et al., 2021b), and gastroparesis (Woodhouse et al., 2018). Consistent with wider literature, the GI-based research has demonstrated that illness perceptions and coping styles influence the relationship between illness symptoms and patient reported outcomes (Bree ). Further, studies have also demonstrated that additions to the CSM through the inclusion of additional known GI-relevant factors (e.g., catastrophising, visceral sensitivity) also add to the CSM’s prediction of illness adjustment outcomes such as QoL (Knowles et al., 2017; Möller et al., 2021b).

Catastrophising has been primarily studied as a maladaptive coping strategy in the adjustment to chronic pain (Hirsh et al., 2007; Ikemoto et al., 2020). Due to pain being a common symptom across GI disorders (Drewes et al., 2020), catastrophising has received some attention in the GI literature. In the GI context, catastrophising can be defined as a tendency to overemphasise the threat value and, social and functional implications of GI-specific sensations and symptoms (Hunt, Ertel, Coello, & *al.*, 2014). Catastrophising has been reported to be predictive of GI symptom severity, GI specific anxiety and QoL in IBS (Hunt et al., 2014, 2018; McKinnon et al., 2015; Sherwin et al., 2016); functional disability and QoL in IBD (De Carlo et al., 2019; Wojtowicz et al., 2014); oesophageal pain sensitivity in gastro-oesophageal reflux disease (GORD; Martel et al., 2016); and to mediate improvement in QoL in cognitive behaviour therapy interventions for IBS (Hunt, Miguez, Dukas, Onwude, & White, 2021).

As identified in recent reviews (Hagger & Orbell, 2021; Hagger et al., 2017), the research to date applying the CSM has been limited to largely cross-sectional studies. As a consequence, true causal processes, and evidence for the dynamic feedback processes that underpin the CSM have not been evaluated. To address these known limitations, Hagger and colleagues (Hagger & Orbell, 2021) have proposed that the testing of the CSM in a dynamic way requires a cross-lagged panel design. In such a design, the CSM constructs are measured on two or more times with cross-lagged (i.e., reciprocal) and time-lagged effects being simultaneously evaluated. Heeding this call, in a recent study, Möller and colleagues (Möller et al., 2021a) conducted the first cross-lagged analysis of the CSM pre and during the COVID-19 pandemic recruiting 674 individuals living with coeliac disease. In brief, the study provided evidence for the dynamic nature of the CSM. Along with the strong time-lagged autoregressive paths for each of the CSM components (e.g., pre-pandemic GI symptoms predicting pandemic GI symptoms), the final model also identified evidence for cross-lagged paths (e.g., pre-pandemic illness perception predicting maladaptive coping, distress and QoL). The current study sought to add to the limited research applying a cross-lagged panel analysis of the CSM in an international GI cohort during the COVID-19 pandemic, while also accounting for perceptions relating to COVID-19.

### Study Aims and Hypotheses

**Fig. 1:**
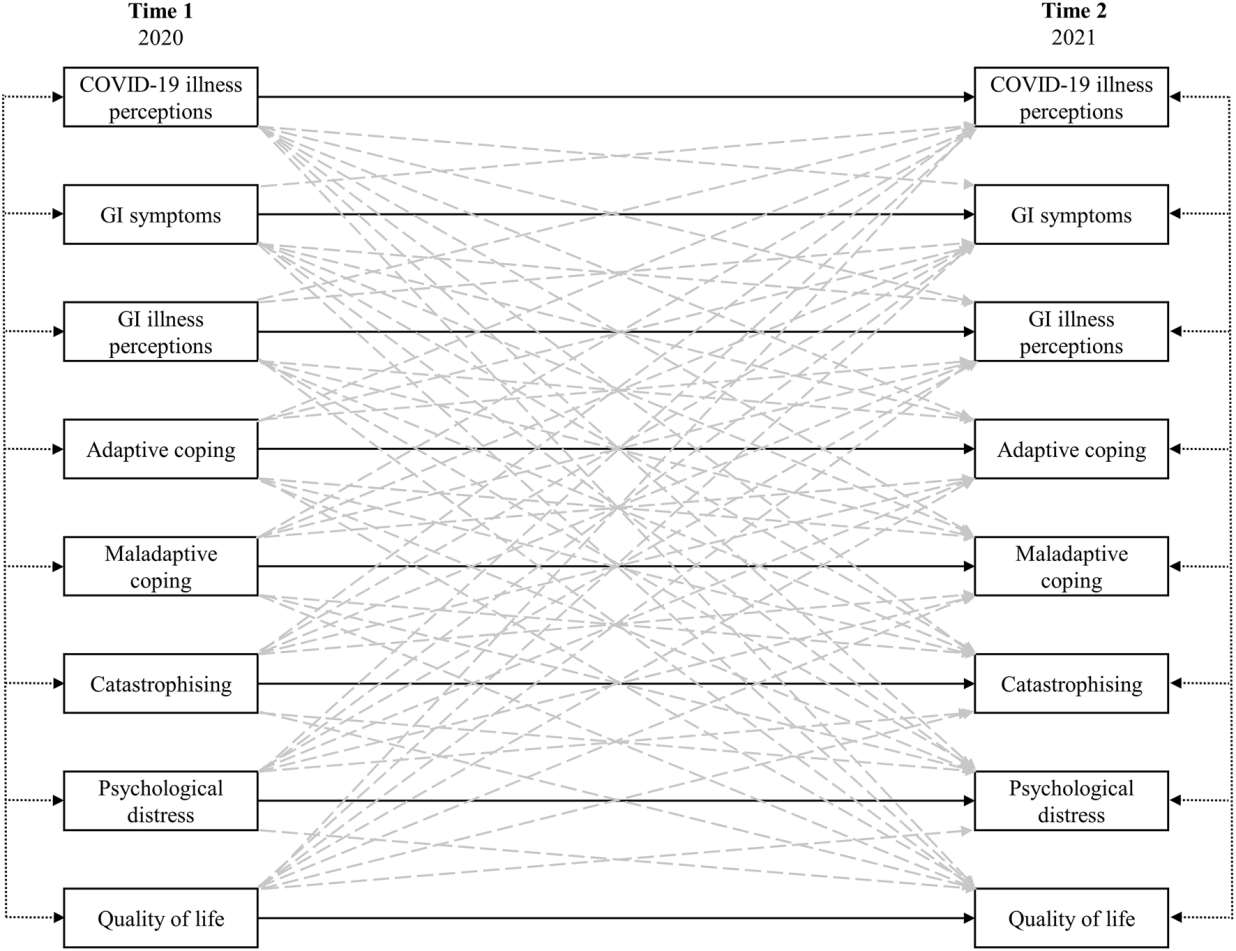
Cross lagged panel mode. Stability (i.e., autoregressive) effects are shown with solid lines. Grey-dashed lines indicate cross-lagged (i.e., reciprocal) effects. For presentation clarity, intercorrelations among variable error terms at their respective time points indicated with double-arrow short-dash black lines

Guided by the CSM and the findings of our recent study (Möller et al., 2021a), we aimed to examine the interrelationships of COVID-19 perceptions, GI symptoms, GI-illness perceptions, coping, catastrophising, psychological distress, and QoL during the COVID-19 pandemic. Utilising a cross-lagged panel model (see Fig. 1), tested via structural equation modelling (SEM), we hypothesised that after controlling for all autoregressive effects and the effects of other predictors:

1. The strongest predictor of a variable at Time 2 would be its Time 1 counterpart (e.g., T1 COVID-19 perceptions—T2 COVID-19 perceptions).
2. COVID-19 perceptions and GI symptoms at Time 1 would predict negative illness perceptions at Time 2.
3. Negative illness perceptions at Time 1 would predict adaptive and maladaptive coping, catastrophising, psychological distress and poorer QoL at Time 2.
4. Maladaptive coping and catastrophising at Time 1 would predict psychological distress and poorer QoL at Time 2.
5. Psychological distress and poor QoL at Time 1 would demonstrate reciprocal (i.e., feedback) effects, predicting COVID-19 perceptions, GI symptoms, negative illness perceptions, maladaptive coping and catastrophising at Time 2.

## Methods

### Procedure

This study is part of an ongoing, international, and longitudinal research project examining the physical and psychological well-being of people with GI disorders during the COVID-19 pandemic. The detailed study protocol for this research project has been previously published (Ferreira et al., 2021). In summary, individuals with a GI condition from different countries (e.g., Australia, UK, USA, Portugal) were invited to participate in an online study via national GI patient organizations and associated social media postings. Ethical approval to conduct this research was obtained from the University Research Ethics Committee and by local ethics committees where required.

Data were collected on two occasions: baseline (28th May—3rd October 2020) and follow-up (19th March—26th May 2021), with an average time between completion of questionnaires being 8.3 months (SD = 1.07). Inclusion criteria included being 18 or older, having a gastrointestinal disorder diagnosis by a physician, and having ability to consent and to communicate in English.

### Measures

#### Gastrointestinal Symptom Severity

The Gastrointestinal Symptom Rating Scale (GSRS; Svedlund et al., 1988) is a well-established measure to assess the severity of symptoms across a range of GI conditions (Dimenäs et al., 1995). The scale consists of 15-items (e.g., “Have you been bothered by NAUSEA?”) assessing a range of gastrointestinal symptoms (e.g., nausea, diarrhea, abdominal pain, indigestion) over the past week. Items are rated on a 7-point Likert scale (1 = “no symptoms” to 7 = “most pronounced symptoms”). Items are summed with a total score ranging from 15 to 105, with higher scores indicating more intense gastrointestinal symptoms. In the current study, the internal consistency of the GSRS at Time 1 and Time 2 were good (0.91, and 0.89 respectively).

#### Gastrointestinal Illness Perceptions

Illness perceptions relating to gastrointestinal conditions were based on a modified Brief Illness Perceptions Questionnaire (BIPQ; Broadbent, Petrie, Main & Weinman, 2006). The 9-item questionnaire exploring emotional and cognitive representations was modified to specifically refer to ‘gastrointestinal illness’, rather than ‘illness’. Consistent with recommendations by Broadbent et al. (2015), a principal axis exploratory factor analysis (EFA) with oblique rotation was conducted to extract a single factor. Four items with a with strong internal consistency (0.88 at Time 1 and Time 2) were identified: ‘How much does your illness affect your life?’, ‘How much do you experience symptoms from your illness?’, ‘How concerned are you about your illness?’, and ‘How much does your illness affect you emotionally?’. Each item was assessed on a 11-point rating scale (0 = “not at all” to 10 = “severely affects my life”), and illness perceptions scores were calculated by averaging the 4 items, with higher scores reflecting a poorer emotional and cognitive representation of illness.

#### COVID-19 Perceptions

To assess the potential influence of the global COVID-19 pandemic, perceptions relating to COVID were evaluated using an adapted version of the 8-iem BIPQ where ‘Illness’ was replaced by ‘COVID-19’ (i.e., “How much does COVID-19 affect your life?”). Principal axis EFA with oblique rotation was used to extract a single factor solution. The seven items retained with excellent internal consistency (0.84 at Time 1 and Time 2) were: “Due to COVID-19, how much does isolation affect you emotionally?”, “How much has COVID-19 affected you emotionally? (e.g., do you feel angry, scared, upset or depressed?)”, “Due to COVID-19, how much do you feel you are isolated?”, “How much does COVID-19 affect your life?”, “How concerned are you about COVID-19?”, “How much does COVID-19 affect your gastrointestinal health?”, and “How long do you think COVID-19 will continue?”. Each item was assessed on a 11-point rating scale (0 = “not at all” to 10 = “severely affects my life”). Perceptions relating to COVID were attained by averaging the seven items, with higher scores indicating poorer emotional and cognitive representation relating to COVID-19.

#### Coping Styles

Coping styles were assessed using the Brief COPE scale (Brief-COPE; Carver, 1997). Consistent with the recommendations by the scale authors, subscales based on the 28-items were derived by using a principal axis EFA with oblique rotation. From the original 28-item questionnaire two subscales were attained: maladaptive coping (5-items) and adaptive coping (7-items). Maladaptive coping had 5 items: ‘I’ve been criticising myself.”, “I’ve been blaming myself for things that happened.”, “I've been giving up the attempt to cope.”, “I've been giving up trying to deal with it.”, and “I've been expressing my negative feelings”, with an internal consistency of 0.74 at Time 1 and 0.73 at Time 2. Adaptive focused coping had 7 items: “I've been concentrating my efforts on doing something about the situation I'm in.”, “I've been trying to come up with a strategy about what to do.”, “I've been taking action to try to make the situation better.”, “I’ve been thinking hard about what steps to take.”, “I’ve been trying to see it in a different light, to make it seem more positive.”, “I've been turning to work or other activities to take my mind off things.”, and “I've been looking for something good in what is happening.” Internal consistency of the adaptive coping subscale was 0.84 at Time 1 and 0.85 at Time 2. Each item is measured on a four-point scale (0 = “I haven’t been doing this at all” to 3 = “I’ve been doing this a lot”), with subscale scores being attained by averaging items; higher scores indicated a greater engagement in maladaptive or adaptive coping.

#### Catastrophising Scale

Catastrophizing was assessed using the catastrophising subscale of the Coping Strategies Questionnaire (CSQ-CAT; Hirsh et al., 2007). The catastrophising subscale consists of 6-items (e.g., “It is terrible and I feel it's never going to get any better.”). Items are rated on a 7-point Likert scale ranging from (0 = “never do that” to 6 = “always do that”). Items are summed with a total score ranging from 0 to 36, with higher scores indicating a more catastrophising about symptoms. In the current study, the internal consistency of the catastrophising subscale at Time 1 and Time 2 were good (0.91 and 0.91 respectively).

#### Psychological Distress

Psychological distress was assessed using the Depression, Anxiety and Stress Scale (DASS-21; Lovibond & Lovibond, 1995). The DASS-21 consists of 21 items (e.g., “I found it hard to wind down”), with 7 items assessing each subscale (depression, anxiety, and stress). Each item is assessed on a 4-point Likert scale ranging from 0 (did not apply to me at all) to 3 (applied to me very much, or most of the time). Item scores are summed and multiplied by two. Total score ranged from 0 to 126, with higher scores indicating greater psychological distress. In the current study, the internal consistency of the DASS-21 at Time 1 and Time 2 were good (0.95 and 0.93 respectively).

#### Quality of Life

Quality of life was measured using the 8-item EUROHIS-QOL (Schmidt et al., 2006) addressing general well-being in the context of goals, expectations, concerns, and societal systems over the past two weeks. Items (e.g., “How would you rate your quality of life?”) are assessed on a 5-point Likert scale ranging from 1 (very dissatisfied) to 5 (very satisfied). Items are summed with a total score ranging from 8 to 40, with higher scores indicating a higher perceived quality of life. In the current study, the internal consistency of the EUROHIS-QOL at Time 1 and Time 2 were good (0.86 and 0.82, respectively).

### Statistical Analyses

The analysis was performed as a cross-lagged autoregressive model predicting psychological and function outcomes through the Common Sense Model, using SPSS and AMOS software. Analysis consisted of three steps. The first step was to examine between-groups and within-groups measurement invariance through MANOVA and chi-square analysis in SPSS. Multivariate analysis of variance (MANOVA) using Wilk’s Lambda was utilised to examine for continuous between-groups Time 1 differences between participants who did and did not complete both surveys and within-groups differences over Time. In the case of significant multivariate effects, the extent of the difference was measured using partial *η*2 and interpreted the size according to Ferguson’s guidelines (Small = 0.04, medium = 0.25, large = 0.64; Ferguson, 2016).

To test the study hypotheses, a saturated cross-lagged autoregressive model was specified with each Time 1 variable predicting the Time 2 variables, and all covariances at each time point being included. To improve parsimony, and consistent with past research (Hwang et al., 2021; Joshanloo, 2018), all cross-lagged pathways that did not achieve statistical significance at *p* < .05 were removed from the saturated model. This trimmed model was then evaluated for model fit using the *χ*2, Comparative Fit Index (CFI), Tucker-Lewis Non-Normed Fit Index (TLI), and the Root Mean Squared Error Approximation (RMSEA). Additionally, any paths that were significant in the saturated model but became non-significant in the trimmed model were also removed. Good model fit is considered achieved when the chi-square for the model is non-significant, the CFI and TLI meet or exceed 0.950, and the RMSEA approaches zero (Hu & Bentler, 1998). Improvements for model fit accounting for change in complexity were evaluated using the Akaike Information Criterion (AIC; Akaike, 1974), and calculating the log-likelihood ratio for significance change (Vrieze, 2012).

## Results

Of the original 831 participants at baseline, 270 (32.5%) completed the follow-up questionnaire. The average age of the Time 1 and Time 2 completers was 47.26 years (SD = 16.53), with the majority being female (85.2%), and having a diagnosis of a gastrointestinal condition for 15.58 years (SD = 14.04). See Table 1 for the full sample demographics at Times 1 and 2.

**Table 1:** Demographic comparison of Time 1-only and Time 1 and Time 2 completers

	Completed time 1 only (*N* = 561)		Completed both Time 1 and Time 2 (*N* = 270)			
	*M*	*SD*	*M*	*SD*	*Sig*	*η*^*2*^
Age	53.34	15.84	47.26	16.53	< .001	0.03
Illness duration	19.69	16.51	15.58	14.04	< .001	0.02

	Count	%	Count	%	*Sig*
Gender					
Male	104	18.5	38	14.1	
Female	454	80.9	230	85.2	
Equally/neither/unsure	1	.17	2	.74	
Other	2	.35	0	0.0	
Marital status					
Single	179	31.9	53	19.6	< .001
Married	298	53.1	166	61.5	.02
Defacto	40	7.1	14	5.2	
Widowed	8	1.4	11	4.1	.02
Divorced	27	4.8	23	8.5	.04
Separated	9	1.6	3	1.1	
Educational attainment					
Elementary school to 8th grade	5	0.9	2	0.7	
Some high school, no diploma	33	5.9	6	2.2	
High school degree or equivalent	62	11.1	22	8.1	.02
Some college credit, no degree	50	8.9	25	9.3	
Trade/technical/vocational training	59	10.5	30	11.1	
Associate degree (e.g., AA, AS)	31	5.5	8	3.0	
Bachelor’s degree (e.g., BA, BS, BBA)	182	32.4	97	35.9	
Master’s degree (e.g., MA, MS, MEng, MSW, MBA)	95	16.9	55	20.4	
Professional degree (e.g., MD, DDS, JD, DVM)	23	4.1	13	4.8	
Doctorate degree (e.g., PhD)	21	3.71	12	4.40	
Current employment					
Full-time employed	203	40.3	67	29.3	.001
Part-time employed	73	13.7	36	14.1	
Casually employed	7	1.6	7	2.2	
Other (please specify):	29	3.7	20	3.3	
Self-employed	32	6.8	17	10.0	
Unemployed	48	4.8	15	2.6	
Retired	87	14.8	66	23.7	.002
Pensioner	28	5.0	20	7.8	
Home duties	23	3.2	15	4.1	
Student	31	3.0	7	6.1	
Global region					
Australia and New Zealand	116	20.6	54	20	
Continental Europe	143	25.4	14	5.1	< .001
Scandinavia	30	5.3	8	2.9	
The Americas	51	9.1	41	15.1	.009
UK and Republic of Ireland	218	38.8	151	55.9	< .001
Other	3	.53	2	.74	
Condition					
Functional only (e.g., irritable bowel syndrome)	80	14.3	34	12.6	
Inflammatory only (e.g., coeliac disease, inflammatory bowel disease)	312	55.7	140	51.9	
Structural only (e.g., Barrett's oesophagus)	21	3.8	5	1.9	
Functional & inflammatory	60	10.7	30	11.1	
Functional & structural	24	4.3	20	7.4	
Inflammatory & structural	31	5.5	26	9.6	.03
Functional, inflammatory, & structural	32	5.7	15	5.6	

Significance tests are Bonferroni corrected

### COVID-19 Context During the Study Collection Periods

During the first data collection period (28th of May—3rd of October, 2020) the global number of active COVID-19 cases increased from 2.7 to 6.7 million, while the rolling 7-day average of daily deaths increased from 5,336 on the 28th of May to a peak of 7582 in mid-late July, before reducing to 4971 by the 3rd of October, when the first collection period ended (Worldometer, 2021). At Time 2 (19th March—26th May 2021) the global number of active COVID-19 cases remained relatively stable (i.e., approximately 14.6 million cases), however, the rolling 7-day average of daily deaths increased from 9,041 to 12,230 per day worldwide (Worldometer, 2021). It is also important to note that during Time 2 vaccination campaigns across all countries were underway, with the number of administered vaccine doses globally rising from 448.3 million to 1.81 billion doses (Roser & Ortiz-Ospina, 2021). In relation to COVID-19 infections at Time 1, 34 (4.1%) participants reported having been infected with COVID, with 11 reporting (1.3%) current symptoms. At Time 2, 7 participants (2.6%) reported having COVID-19 since the T1, with only 1 (0.4%) reporting current symptoms. MANOVA involving the study dependant variables revealed that only COVID-19 perceptions at Time 1 and Time 2 were significantly higher for those with a history of COVID-19 compared to those without. Additionally, participants in this study were asked about their level of social isolation (see Table 2). Significantly fewer participants were engaging in no social isolation (i.e., going out and not engaging in social distancing) from baseline (Time 1) to follow-up (Time 2). Most participants in both data collection periods were engaging in moderate social isolation (i.e., staying at home and only going out for food and engaging in social distancing) or engaging in limited social isolation (i.e., mostly staying at home, but going out for food and seeing friends/family).

**Table 2:** Sample COVID-19 social isolation status across Time 1 and Time 2

	Time 1 *n* (%)	Time 2 *n* (%)	*Sig*
Total isolation due to having COVID-19 symptoms	1 (.4)	0 (0)	–
Strict social isolation due to mandatory quarantine	3 (1.1)	3 (1.1)	1
Strict social isolation (i.e., staying at home and not going out at all)	16 (5.9)	17 (6.3)	.857
Moderate social isolation (i.e., staying at home and only going out for food and engaging in social distancing)	108 (40.0)	144 (53.3)	.002
Limited social isolation (i.e., mostly staying at home, but going out for food and seeing friends/family)	132 (48.9)	76 (28.1)	< .001
No social isolation (i.e., going out and not engaging in social distancing)	10 (3.7)	30 (11.1)	< .001

*N* = 270

Participants who only completed surveys during Time 1 (Time 1 only participants) were slightly older and reported longer illness durations compared to those who completed both Time 1 and Time 2 surveys (Time 2 completers). Time 2 completers were more likely to be single, widowed, or divorced, while Time 1 only participants were more likely married. Education levels were broadly equal between subsamples. Time 1 only participants were more likely to be in full time employment, while Time 2 completers were more likely to be retired. Time 1 only participants were more likely to be from continental Europe, while Time 2 completers were more likely to be from North or South America, the UK, and Republic of Ireland. Finally, Time 1 only participants were marginally less likely to report having both a structural and functional GI condition. Despite statistical significance, most differences are small or trivial in magnitude.

A MANOVA revealed overall significant multivariate effects between Time 1 only and Time 2 completing participants (Box’s M = 44.74, *p* = .164; Wilks Lambda = 0.980, F (8, 822) = 2.087, *p* = .04, *η*^2^ = 0.020). Participants that completed Time 1 only reported greater GI symptoms and reduced QoL compared to those that completed follow-up, however, effect sizes were very small (See Table 3). Moreover, Levene’s test of equality of error variances indicated that homogeneity of variances could not be assumed for GI symptoms. Given the small magnitude of effect for GI symptoms, no further tests were performed.

**Table 3:** Time 1 outcome variables compared between Time 2 completers and non-completers

	Time 1 only (*N* = 561)		Time 2 completers (*N* = 270)			
	*M*	*SD*	*M*	*SD*	*Sig*	*η*^*2*^
COVID-19 perceptions	5.31	2.02	5.39	1.93	.58	
GI symptoms	37.00	17.22	34.39	15.00	.03	.005
GI illness perceptions	5.92	2.43	5.57	2.50	.05	
Adaptive coping	2.35	0.69	2.36	0.70	.76	
Maladaptive coping	1.65	0.57	1.61	0.53	.34	
Catastrophising	8.98	8.32	8.86	8.01	.84	
Psychological distress	27.34	26.01	26.13	24.12	.52	
Quality of life	28.20	6.23	29.38	5.81	.009	.008

### Change in Model Variables from Pre-pandemic to Pandemic

A one-way within-subjects MANOVA identified a statistically significant overall effect between the Time 1 and Time 2 variables (Wilks Lambda = 0.918, F (8, 262) = 2.92, *p* = .004, *η*^2^ = 0.08). As shown in Table 4, compared to Time 1 measures, participants reported small, yet statistically significant reductions in negative GI illness perceptions, adaptive coping styles, and catastrophising. In contrast, there were no statistically significant differences between timepoints for GI symptoms, maladaptive coping strategies, psychological distress, or quality of life scores.

**Table 4:** Outcome measures over time

	Time 1		Time 2			
	*M*	*SD*	*M*	*SD*	*Sig*	*Eta*^*2*^
COVID-19 perceptions	5.39	1.93	5.31	1.91	.35	.003
GI symptoms	34.39	15.00	34.17	14.07	.71	.001
GI illness perceptions	5.57	2.50	5.24	2.36	< .001	.040
Adaptive coping	2.36	0.70	2.27	0.66	.01	.024
Maladaptive coping	1.61	0.53	1.58	0.53	.31	.004
Catastrophising	8.86	8.01	7.83	7.81	.007	.027
Psychological distress	26.13	24.12	26.10	20.63	.98	< .001
Quality of life	29.38	5.81	29.43	5.34	.86	< .001

### Correlations Across the Time 1 and Time 2 Study Variables

As shown in Table 5, almost all within-timepoint correlations were statistically significant, with two exceptions: quality of life was found to be unrelated to adaptative coping at Time 1 and 2. Overall, adaptive coping was found to be the weakest correlate of all variables in the model.

**Table 5:** Correlations across the Time 1 and Time 2 study variables

	1	2	3	4	5	6	7	8
1. COVID-19 perceptions		.41^***^	.52^***^	.24^***^	.38^***^	.38^***^	.51^***^	− .38^***^
2. GI symptoms	.39^***^		.71^***^	.28^***^	.42^***^	.44^***^	.54^***^	− .43^***^
3. GI illness perceptions	.51^***^	.64^***^		.21^***^	.40^***^	.48^***^	.52^***^	− .44^***^
4. Adaptive coping	.21^***^	.12^***^	.17^***^		.26^***^	.21^***^	.19**	− .09
5. Maladaptive coping	.38^***^	.31^***^	.37^***^	.24^***^		.68^***^	.70^***^	− .49^***^
6. Catastrophising	.48^***^	.39^***^	.52^***^	.13^***^	.68^***^		.73^***^	− .46^***^
7. Psychological distress	.55^***^	.48^***^	.48^***^	.13^***^	.69^***^	.73^***^		− .50^***^
8. Quality of life	− .45^***^	− .47^***^	− .51^***^	.01	− .48^***^	− .58^***^	− .60^***^	

Correlations below the diagonal are correlations between T1 predictors, correlations above are between T2 outcomes

***p < .001 **p < *.01*

### Cross-Lagged Panel Model

**Fig. 2:**
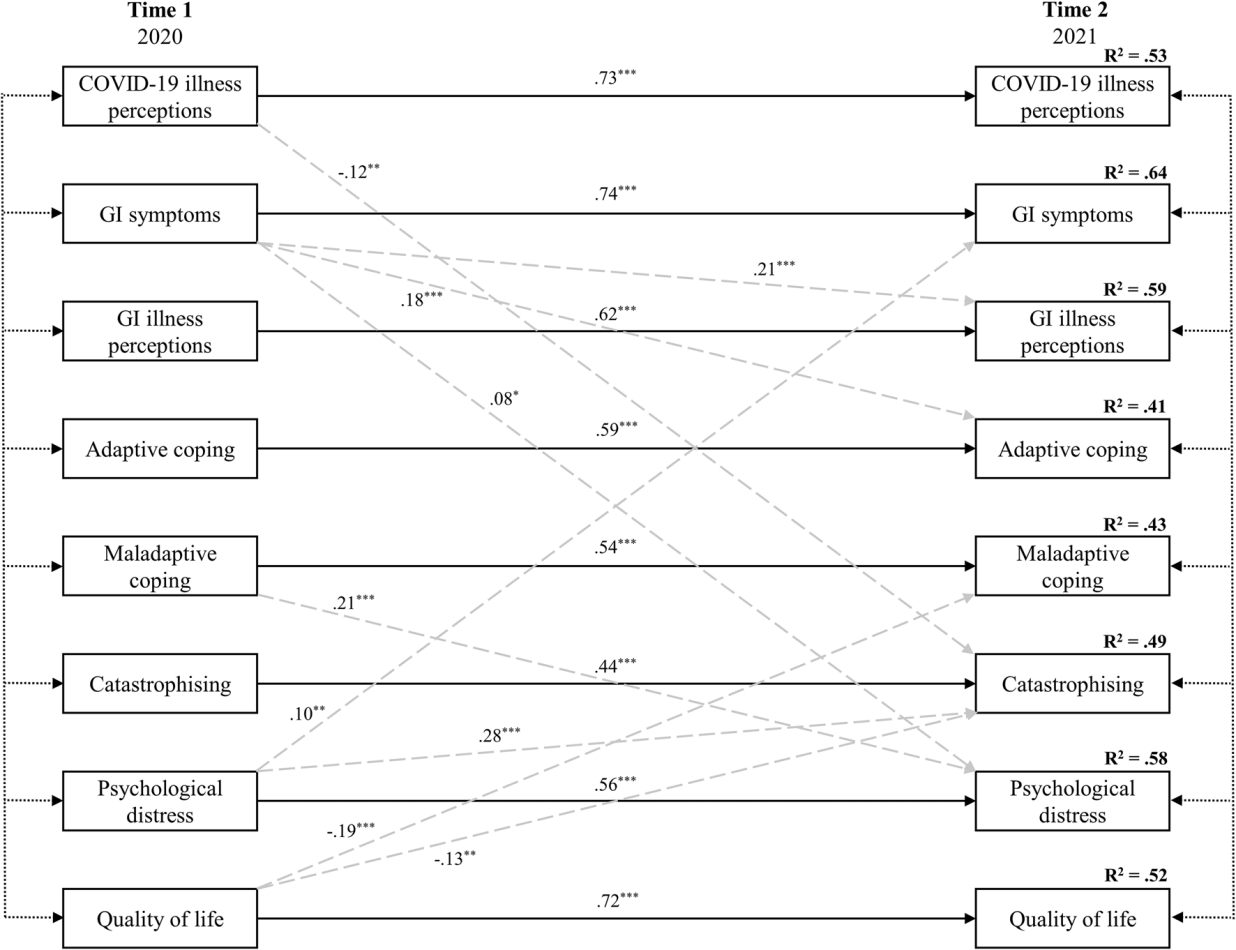
Final cross-lagged panel model after removing non-significant paths. Time 1: *n* = 831, Time 2: *n* = 270. Stability (i.e., *autoregressive*) effects are shown with solid lines and path coefficients aligned directly down the middle of the figure. Grey-dashed lines indicate cross-lagged (i.e., reciprocal) effects. For presentation clarity, intercorrelations among variable error terms at their respective timepoints are indicated with double-arrow short-dash black lines

A two-wave fully saturated cross-lagged panel model was specified to test autoregressive effects for model outcomes, adjusted by the inclusion of complete cross-lagged effects over time and all contemporaneous correlations (see Appendix A for the saturated cross-lagged model results prior to post-hoc modifications). Post-hoc modifications by removing non-significant cross-lagged effects resulted in a final model that fitting the data extremely well (*Χ*^2^(47) = 62.80, *p* = .06. CFI = 0.997. TLI = 0.991. RMSEA = 0.020). The AIC of the trimmed model was 272.80 (a reduction of 31.20; *p* < .(.001), also suggested that the final model (see Fig. 2) was a significant improvement on the original; see Table 5 for the final model parameter estimates.

As shown in Fig. 2 and Table 6, the removal of non-significant regression pathways substantially simplified the model, while only resulting in minimal reduction in endogenous variance. The two largest changes in *R*^2^ were for Time 2 GI illness perceptions (-5.6%) and Time 2 catastrophising (-4.7%), but the *R*^2^ remained substantial after the reduction of six and four predictive pathways, respectively. In all outcomes, a large proportion of variance was explained. The final model accounted for 63.7% of the variance in Time 2 GI symptoms, 58.8% of Time 2 GI illness perceptions, 40.5% of Time 2 adaptive coping, 43.2% of Time 2 maladaptive coping, 49.4% of Time 2 catastrophising, 58.2% of Time 2 psychological distress, 51.8% of Time 2 quality of life, and 52.9% of Time 2 COVID-19 perceptions.

**Table 6:** Parameter estimates for final cross-lagged model showing unstandardised estimates, standard errors in parentheses, followed by critical ratios, and standardised estimates

	T2 COVID-19 perceptions	T2 GI symptoms	T2 GI illness perceptions	T2 Adaptive coping	T2 Maladaptive coping	T2 Catastro-phising	T2 Psychological distress	T2 Quality of life
T1 COVID-19 perceptions	0.69 (0.04) 18.31*** 0.73					− 0.46 (0.17) − 2.71** − 0.12		
T1 GI symptoms		0.65 (0.03) 19.21*** 0.74	0.03 (0.01) 4.50*** 0.21	0.01 (0.002) 3.86*** 0.18			0.09 (0.04) 2.08* 0.08	
T1 GI illness perceptions			0.57 (0.04) 14.19*** 0.62					
T1 Adaptive coping				0.56 (0.04) 12.91*** 0.59				
T1 Maladaptive coping					0.51 (0.04) 12.09*** 0.54		7.57 (1.61) 4.69*** 0.21	
T1 Catastrophising						0.41 (0.05) 8.05*** 0.44		
T1 Psychological distress		0.06 (0.02) 2.79** 0.10				0.08 (0.02) 4.72*** 0.28	0.45 (0.04) 11.7*** 0.56	
T1 Quality of life					− 0.02 (0.004) -4.25*** − .19	− 0.16 (0.06) − 2.70** − 0.13		0.63 (0.04) 17.97*** 0.72

Time 1: *n* = 831, Time 2: *n* = 270. Empty cells indicate paths removed due to non-significance. *GI* Gastrointestinal

***p < .001, **p < .01, *p < .05

The results indicated that hypothesis 1 was fully supported, as each autoregressive pathway from Time 1 to Time 2 was consistently the strongest predictor of each outcome, and positive in direction (See Table 5). Hypothesis 2 was largely supported. GI illness perceptions at Time 2 were strongly predicted by Time 1 GI illness perceptions, and to a lesser extent, Time 1 GI symptoms. However, COVID-19 perceptions at Time 2 were only predicted by Time 1 COVID-19 perceptions. Hypothesis 3 was unsupported, as perceptions for both GI illness and COVID-19 were unrelated to any of adaptive and maladaptive coping, catastrophising, psychological distress or quality of life in the final model. Likewise, hypothesis 4 was unsupported. Psychological distress and quality of life at Time 2 were predicted only by their Time 1 counterparts. Finally, hypothesis 5 was partially supported. Psychological distress at Time 1 was predictive of GI symptoms and catastrophising at Time 2, but not COVID-19 perceptions or maladaptive coping. Quality of life at Time 1 was predictive of maladaptive coping and catastrophising at Time 2, but not COVID-19 perceptions or GI symptoms.

## Discussion

Despite the frequency and diversity in which the CSM has been applied across chronic illness conditions, its application has been largely limited to cross-sectional studies, limiting evidence for its purported dynamic processes. To explore the dynamic processes of the CSM, several researchers have called for the evaluation of the CSM using cross-lagged panel model approaches using longitudinal-based studies (Hagger & Orbell, 2021; Hagger et al., 2017). To the authors knowledge only one study (Möller et al., 2021a) has applied a cross-lagged panel design to evaluate the CSM. Guided by the findings of Möller et al. (Möller et al., 2021a), using a cross-lagged panel design, we aimed to examine the interrelationships of COVID-19 perceptions, GI symptoms, GI-illness perceptions, coping, catastrophising, psychological distress, and QoL during the COVID-19 pandemic.

Hypothesis one was supported, with each measure at Time 2 being most strongly predicted by its Time 1 counterpart. These findings are consistent with the recent CSM-guided cross-lagged coeliac study of (Möller et al., 2021a), which found the autoregression effects of GI symptoms, illness perceptions, maladaptive coping, psychological distress, and QoL to be the strongest time-lagged relationships. Hypothesis two was largely supported. As with (Möller et al., 2021a), GI Illness perceptions at Time 2 were strongly predicted by Time 1 GI illness perceptions, and to a lesser extent, T1 GI symptoms. These findings provide longitudinal evidence for the reliable relationship between GI symptoms and illness perceptions seen in cross-sectional CSM-based GI studies (Bree Hayes et al., 2021; Hayes et al., 2021; Kantidakis et al., 2021; Knowles et al., 2017; Knowles et al., 2011; Möller et al., 2021b; Woodhouse et al., 2018; Zhang et al., 2016). However, contrary to hypothesis 2, COVID illness perceptions at Time 2 were only predicted by Time 1 COVID illness perceptions.

Hypothesis three was unsupported. Time 1 illness perceptions for both GI and COVID were only predictive of their Time 2 counterparts. Our findings stand in contrast to the coeliac cross-lagged panel model study of Möller et al. (Möller et al., 2021a), wherein GI illness perceptions demonstrated the greatest predictive utility in their model, predicting GI symptoms, psychological distress, and QoL over time. Unexpectedly, more negative COVID illness perceptions predicted reduced catastrophising across time and our results did not support previous cross-sectional GI cohort studies finding negative GI illness perceptions to be associated with pain catastrophising (Möller et al., 2021b), and maladaptive coping, psychological distress and reduced QoL (Knowles et al., 2013, 2017; Möller et al., 2021b; Woodhouse et al., 2018; Zhang et al., 2016).

Hypothesis four was unsupported, with Time 2 psychological distress and QoL only being predicted by their Time 1 counterparts. These findings do not support previous cross-sectional GI cohort studies finding psychological distress and/or poor QoL to be associated with maladaptive coping (Knowles et al., 2013, 2017; Woodhouse et al., 2018) and pain catastrophising (Cassar et al., 2018; Möller et al., 2021b). Our findings were, however, consistent with Möller et al. (Möller et al., 2021b) insofar as neither maladaptive coping nor pain catastrophising demonstrated significant time-lagged effects on well-being outcomes (i.e., psychological distress, QoL). Finally, hypothesis five was partially supported. Consistent with the hypothesis and Möller et al. (Möller et al., 2021a), psychological distress at Time 1 was predictive of GI symptoms and catastrophising at Time 2. Psychological distress did not predict COVID illness perceptions as was hypothesised, nor did distress predict maladaptive coping across time, as was found by Möller et al. (Möller et al., 2021a). Consistent with prediction and in contrast to Möller et al. (Möller et al., 2021b), Time 1 QoL was predictive of T2 maladaptive coping and catastrophising. Further, QoL was not predictive of Time 2 COVID or GI illness perceptions, nor did QoL predict GI symptoms across time as found by Möller et al. (Möller et al., 2021a).

As identified above, several hypotheses were not supported (i.e., 2, 3, and 4) suggesting that some of the CSM variables at Time 1 (i.e., GI illness perceptions, adaptive coping) had limited utility in predicting other variables at Time 2 beyond their Time 2 counterparts. These findings suggest that overall gastrointestinal symptoms, covid- and illness-related perceptions, psychological distress and QoL remained stable. This stability may have been due to the fact that on average participants had been living with their GI condition for 16 years, hence their GI diseases might have been quite stable and more resistant to extrinsic stresses, such as the COVID-19 pandemic. However, and importantly, the associations across these variables at each time point were both strongly related and highly significant. The strength of the results may indicate that in this study, the influence of some Time 2 variables on other Time 2 variables (e.g., illness perceptions on coping, illness perceptions and coping on psychological distress/QoL) are better explained through present rather than past time effects (Time 1 > Time 2). Unlike Möller et al. (Möller et al., 2021a), the lack of prediction across Time 1 and Time 2 variables, may also be in part due to the study design. Möller and colleagues study (Möller et al., 2021a) involved assessing the CSM based on data from a large coeliac cohort collected both prior to and during the pandemic. It is possible that the current findings reflect the limited changes from Time 1 and Time 2, compared to that found by Möller et al. (Möller et al., 2021b).

### Limitations and Future Research

While this study is one of the first to apply cross-lagged panel model using longitudinal-based design to evaluate the CSM, it is not without limitations. Although based on an international collaboration, the sample size, while moderately large for a longitudinal design, was not necessarily representative of the prevalence rates of specific GI conditions. Further, participation was also limited to those willing to complete multiple long questionnaires and being fluent in English. This selection bias is reflected in the demographic characteristic of the sample with around 85% of participants being female and having an average GI condition diagnosis duration of 16 years. Another notable limitation was heterogeneity of GI conditions included in the study and the subsequent need to use a validated generic symptom scale, rather than condition specific measures, including validated biological markers of condition severity (e.g., faecal calprotectin for inflammatory bowel disease activity). While the COVID-19 pandemic had a substantial on society and individuals generally, across the duration of the study, less than 5% of participants reported having COVID-19. Given that there was a relatively small number of participants who had experienced COVID, exploring the impact of this in the model was not possible.

As noted by others (Hagger et al., 2017; Hagger & Orbell, 2021; B. ; Leventhal et al., 2016), the CSM provides a valuable model to explore the dynamic interplay of psychosocial processes relating to illness adjustment. However, it is also clear that future research not only needs to extend the CSM but also the way in which it is assessed and validated. In relation to extending the model, researchers should look to include other well-established health models (e.g., theory of planned behaviour) and how they may influence intentions and actions relating to illness adjustment (Hagger & Orbell, 2021). Researchers should also seek to extend the CSM by exploring the potential role of biological measures (e.g., faecal calprotectin) and relevant illness-specific factors, for example visceral sensitivity and pain catastrophising (e.g., Gastrointestinal Unhelpful Thoughts Scale [GUTS] Knowles et al., 2022) in GI-based studies (B. ). While the evaluation of the CSM using longitudinal cross-lagged approaches is important, it is also important to acknowledge that this approach may not be sensitive to the quick changes in the variables (Hagger & Orbell, 2021; B. ), and that questionnaire-based studies may also not be inadequate in capturing the dynamic character of the CSM (Leventhal et al., 2016). Given this, future studies should explore the potential value of evaluating the CSM using a mixed-method approaches, which could include ecological momentary assessment (Breland et al., 2020) to fully explore the CSM's dynamic nature.

### Clinical Implications

The adjustment to illness and its inherent impact on psychological well-being and quality of life is influenced by several psychosocial processes. Consistent with cross-sectional and longitudinal based applications of the CSM involving GI cohorts (Bree Hayes et al., , 2021; Knowles et al., 2017; Möller et al., 2021a, 2021b; Woodhouse et al., 2018), modifiable psychosocial processes such as illness perceptions and coping styles, particularly maladaptive forms, would be important to identify and target in psychological interventions. Also reflecting past GI-based research (Hunt et al., 2014), this study highlights the role of catastrophising as an important process to identify and target through psychological intervention. Such therapeutic targeting through cognitive behaviour therapy or acceptance and commitment therapy is likely to be more impactful by identifying and addressing underlying pathogenic processes known to impact psychopathology (e.g., rumination and avoidance; Barlow & Barlow, 2018).

## Conclusion

Based on an international gastrointestinal cohort, this study evaluated an extended CSM across two points during the COVID-19 pandemic using a cross-lagged panel design. This novel approach addresses the limitations of traditional cross-sectional based CSM studies and provides evidence for the dynamic interplay between the CSM components over time. Overall, the findings were mixed in relation to the study hypotheses. Compared to Time 1 measures, only small significant reductions in negative illness perceptions, adaptive coping styles, and catastrophising were reported. These findings suggest that overall GI symptoms, COVID- and illness- related perceptions, psychological distress and QoL remained stable and may have been due to the average diagnosis of 20 years, and therefore more resistant to extrinsic stresses, such as the pandemic. Providing support for the CSM was the finding that the cross-lagged model demonstrated strong time-lagged autoregressive paths for each of the study measures (e.g., Time 1 GI symptoms predicting Time 2 GI symptoms).

As purported by the CSM, the final model also identified evidence for multiple cross-lagged paths (e.g., Time 1 GI symptoms predicting Time 2 illness perceptions, Time 1 maladaptive coping predicting Time 2 psychological distress and catastrophising) and the important role of the CSM variables (e.g., illness perceptions, adaptive and maladaptive coping) and catastrophising influencing psychological distress and QoL within each time point. From a theoretical perspective the study has provided an important contribution to the ongoing application of the CSM to explore, and understand, the complex interplay of psychosocial processes underpinning illness adaptation. At a practical level, the study highlights the importance of identifying and targeting psychosocial processes (such as illness perceptions, coping and catastrophising) in order to promote illness adaptation and in turn increase psychological well-being and QoL.

## Data Availability

The summary data generated during and/or analysed during the current study are available from the corresponding author on reasonable request and after relevant ethical approval.

## Appendix: Model estimates for saturated cross-lagged model

**Table:**

T1 Predictor	*B*	SE	βeta	*Z*	*p*
GI Symptoms					
Adaptive coping	1.327	0.803	1.652	0.061	0.098
Catastrophising	0.067	0.104	0.647	0.037	0.517
Psychological distress	0.119	0.036	3.315	0.203	< 0.001
Psychological distress	0.622	0.042	14.662	0.692	< 0.001
Covid illness perceptions	− 0.105	0.336	− 0.311	− 0.014	0.756
GI illness perceptions	0.601	0.309	1.947	0.099	0.052
Maladaptive coping	− 3.851	1.399	− 2.752	− 0.145	0.006
Quality of life	0.105	0.117	0.901	0.043	0.367
*R*^*2*^ = *0.68*					
GI Illness perceptions					
Adaptive coping	− 0.053	0.134	− 0.393	− 0.015	0.694
Catastrophising	0.024	0.017	1.374	0.082	0.169
Psychological distress	0.008	0.006	1.312	0.084	0.189
Psychological distress	0.026	0.007	3.669	0.182	< 0.001
Covid illness perceptions	0.070	0.056	1.241	0.058	0.215
GI illness perceptions	0.579	0.052	11.213	0.600	< 0.001
Maladaptive coping	− 0.559	0.234	− 2.388	− 0.132	0.017
Quality of life	0.000	0.020	0.002	0.000	0.998
*R*^*2*^ = *0.64*					
Adaptive coping					
Adaptive coping	0.547	0.048	11.455	0.570	< 0.001
Catastrophising	0.005	0.006	0.750	0.057	0.453
Psychological distress	− 0.003	0.002	− 1.355	− 0.111	0.175
Psychological distress	0.009	0.003	3.451	0.218	< 0.001
Covid illness perceptions	0.001	0.020	0.059	0.004	0.953
GI illness perceptions	0.003	0.018	0.156	0.011	0.876
Maladaptive coping	0.083	0.083	0.994	0.070	0.320
Quality of life	0.002	0.007	0.232	0.015	0.817
*R*^*2*^ = *0.42*					
Maladaptive coping					
Adaptive coping	− 0.026	0.037	− 0.711	− 0.034	0.477
Catastrophising	0.008	0.005	1.712	0.125	0.087
Psychological distress	0.000	0.002	− 0.100	− 0.008	0.921
Psychological distress	0.004	0.002	1.985	0.119	0.047
Covid illness perceptions	0.015	0.016	0.968	0.055	0.333
GI illness perceptions	− 0.005	0.014	− 0.345	− 0.022	0.730
Maladaptive coping	0.437	0.065	6.728	0.453	< 0.001
Quality of life	− 0.012	0.005	− 2.202	− 0.135	0.028
*R*^*2*^ = *0.48*					
Catastrophising					
Adaptive coping	0.123	0.509	0.242	0.011	0.809
Catastrophising	0.418	0.066	6.356	0.433	< 0.001
Psychological distress	0.071	0.023	3.103	0.226	0.002
Psychological distress	0.015	0.027	0.540	0.030	0.589
Covid illness perceptions	− 0.464	0.213	− 2.176	− 0.116	0.030
GI illness perceptions	0.228	0.196	1.164	0.071	0.244
Maladaptive coping	0.607	0.888	0.683	0.043	0.494
Quality of life	− 0.166	0.074	− 2.246	− 0.128	0.025
*R*^*2*^ = *0.54*					
Psychological distress					
Adaptive coping	− 0.718	1.233	− 0.583	− 0.023	0.560
Catastrophising	0.203	0.159	1.274	0.079	0.203
Psychological distress	0.416	0.055	7.562	0.499	< 0.001
Psychological distress	0.135	0.065	2.066	0.105	0.039
Covid illness perceptions	0.488	0.516	0.947	0.046	0.344
GI illness perceptions	0.206	0.474	0.434	0.024	0.664
Maladaptive coping	5.508	2.149	2.564	0.146	0.010
Quality of life	− 0.138	0.179	− 0.768	− 0.040	0.443
*R*^*2*^ = *0.62*					
Quality of life					
Adaptive coping	− 0.193	0.350	− 0.551	− 0.024	0.582
Catastrophising	0.009	0.045	0.191	0.013	0.849
Psychological distress	0.012	0.016	0.742	0.054	0.458
Psychological distress	− 0.011	0.018	− 0.582	− 0.032	0.560
Covid illness perceptions	0.039	0.146	0.266	0.014	0.790
GI illness perceptions	− 0.177	0.135	− 1.317	− 0.079	0.188
Maladaptive coping	0.249	0.610	0.408	0.025	0.683
Quality of life	0.662	0.051	13.023	0.738	< 0.001
*R*^*2*^ = *0.55*					
COVID Illness perceptions					
Adaptive coping	− 0.027	0.121	− 0.227	− 0.010	0.820
Catastrophising	− 0.012	0.016	− 0.750	− 0.049	0.453
Psychological distress	0.007	0.005	1.303	0.092	0.193
Psychological distress	0.009	0.006	1.372	0.074	0.170
Covid illness perceptions	0.630	0.050	12.488	0.644	< 0.001
GI illness perceptions	0.057	0.046	1.223	0.072	0.221
Maladaptive coping	− 0.091	0.210	− 0.432	− 0.026	0.666
Quality of life	− 0.012	0.018	− 0.693	− 0.038	0.488
*R*^*2*^ = *0.57*					
